# Effects of estrogen and mechanical loading on cultured cells derived from mandibular condylar cartilage

**DOI:** 10.1038/s41598-025-07770-4

**Published:** 2025-07-02

**Authors:** Jin Tsuchida, Yuya Nakao, Koken Sato, Osamu Uehara, Itaru Mizoguchi, Yoshihiro Abiko, Masahiro Iijima

**Affiliations:** 1 Division of Orthodontics and Dentofacial Orthopedics, Department of Oral Growth and Development, School of Dentistry, Health Sciences University of Hokkaido, 1757 Kanazawa, Ishikari−Tobetsu, Hokkaido 061−0293 Japan; 2 Division of Disease Control and Molecular Epidemiology, Department of Oral Growth and Development, School of Dentistry, Health Sciences University of Hokkaido, 1757 Kanazawa, Ishikari−Tobetsu, Hokkaido 061−0293 Japan; 3 Division of Orthodontics and Dentofacial Orthopedics, Tohoku University Graduate School of Dentistry, 4-1 Seiryo-Machi, Aoba-Ku, Sendai, 980-8575 Japan; 4 Division of Oral Medicine and Pathology, Department of Human Biology and Pathophysiology, School of Dentistry, Health Sciences University of Hokkaido, 1757 Kanazawa, Ishikari−Tobetsu, Hokkaido 061−0293 Japan

**Keywords:** Temporomandibular joint osteoarthritis, Mandibular condylar cartilage, Estrogen, Mechanical loading, Extracellular matrix, Sequencing, Gonadal hormones

## Abstract

Temporomandibular joint osteoarthritis (TMJ-OA) is a degenerative condition influenced by mechanical loading and hormonal factors, particularly estrogen. Despite its established role in TMJ pathophysiology, the mechanisms underlying the effects of estrogen on joint cartilage remain unclear, with conflicting reports regarding its protective and destructive effects. This study aimed to investigate the molecular effects of estrogen and mechanical loading on cultured cells derived from mandibular condylar cartilage (MCC). The chondrogenic identity of cells was confirmed using quantitative reverse transcription PCR (qRT-PCR). The cells were then subjected to mechanical loading with or without 17β-estradiol treatment. RNA sequencing and bioinformatics analyses, including differential gene expression, Gene Ontology, and protein–protein interaction network analysis, identified extracellular matrix (ECM)-related pathways and hub genes, such as COL1A1, COL3A1, LAMC1, and LAMB1, as key mediators. Mechanical loading reduced the expression of ECM-related genes in MCC cells, whereas estrogen combined with loading significantly upregulated COL1A1 and COL3A1 expression, as confirmed by qRT-PCR and western blotting. This study demonstrated the effects of estrogen on mechanical loading using superficial condylar cells to elucidate the mechanisms of TMJ. Estrogen may be, at least in part, significantly involved in TMJ-OA via altering of the ECM levels such as collagen, laminin, MMPs, and ADAMTS.

**Supplementary Information:**

The online version contains supplementary material available at 10.1038/s41598-025-07770-4.

## Introduction

Osteoarthritis of the temporomandibular joint (TMJ-OA) is a degenerative condition characterized by destructive bony changes in the mandibular condyle and the joint tissues. When TMJ-OA progresses bilaterally, the mandible rotates clockwise, resulting in severe skeletal Class II malocclusion with an anterior open bite^1,2^. In contrast, unilateral involvement of TMJ-OA causes mandibular deviation toward the affected side, leading to facial asymmetry. The mandibular condylar cartilage contains high-molecular-weight matrices, such as versican, aggrecan, and types I and II collagens, which provide viscoelastic properties to withstand stretching and compressive forces and lubrication against friction. TMJ-OA is proposed to be caused by an imbalance between environmental and host factors, with mechanical loads from parafunction, chronic trauma, and malocclusion cited as environmental factors^3,4^. Excessive mechanical load on the joint surface often disrupts the metabolic balance of the joint cartilage, leading to cartilage cell death, extracellular matrix (ECM) breakdown, and subchondral bone remodeling.

Epidemiological surveys of TMJ-OA have reported that the male-to-female ratio of patients seeking treatment ranges from 1:3 to 1:9, with a higher prevalence among women^5–8^. Moreover, although TMJ-OA is common in older adults, severe conditions are typically observed in young women with unstable estrogen levels or postmenopausal women, displaying a bimodal disease pattern^9,10^. Experimentally induced TMJ-OA is exacerbated by estrogen^11^, leading to joint destruction^12^. However, several other studies have reported a protective effect of estrogen on joint cartilage^13–15^. The effects of estrogen on joint cartilage are inconsistent, and the underlying mechanisms remain unclear. Therefore, a comprehensive analysis is necessary to elucidate the impact of estrogen, which is crucial for TMJ development. Although OA − related molecules have been detected through genome-wide analysis using knee cells, TMJ-derived cells have not yet been used for such an analysis. Given that the types of estrogen receptors differ between the knee and TMJ, the effects of estrogen on each joint vary^16,17^.

This study aimed to recreate the loading conditions of the temporomandibular joint using cultured MCC cells to comprehensively observe the effects of estrogen, and particularly to identify molecules that may provide clues to the mechanisms of TMJ-OA development.

## Results

### mRNA expression analysis of MCC cells

**Fig. 1:**
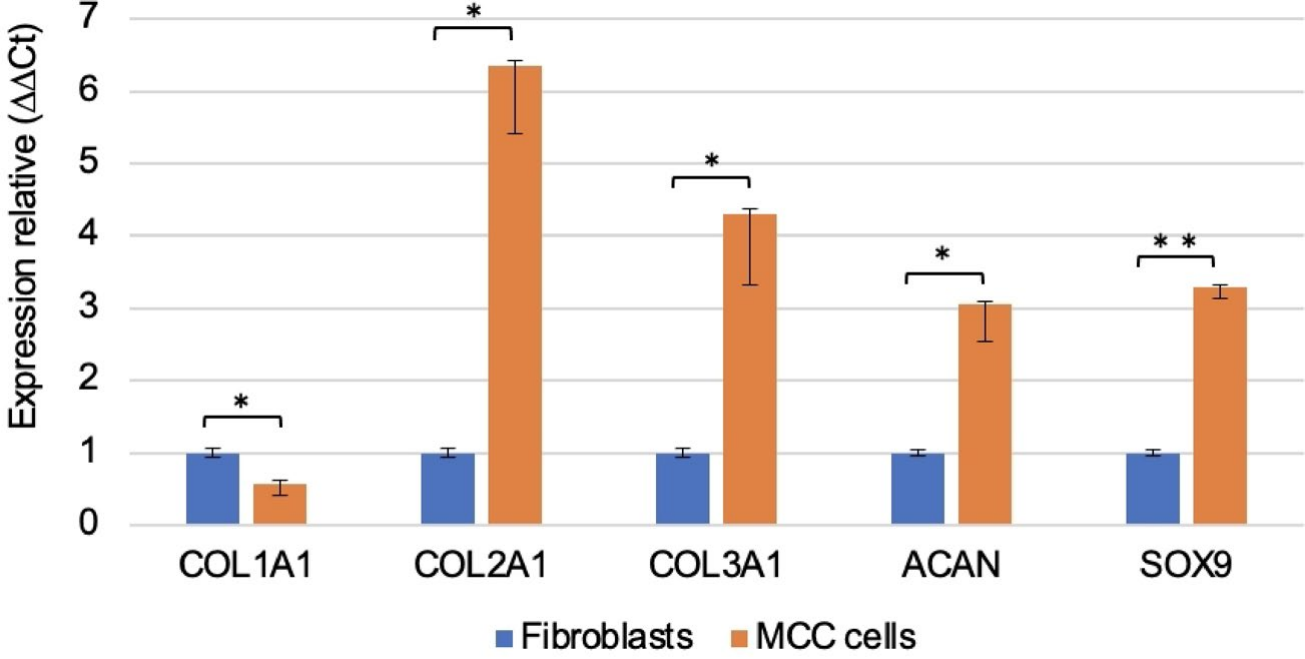
Characterization of MCC cells. Comparison of mRNA expression of each gene in mandibular condylar surface-cultured cells and fibroblasts (n = 6, mean ± standard deviation (SD), *p < 0.05, **p < 0.01). Gene expression was analyzed using the comparative Ct (ΔΔCt) method and normalized to GAPDH expression. In mandibular condylar surface-cultured cells, COL1A1 expression was significantly decreased compared to that in fibroblasts, whereas COL2A1, COL3A1, ACAN, and SOX9 expression was significantly increased.

**Fig. 2:**
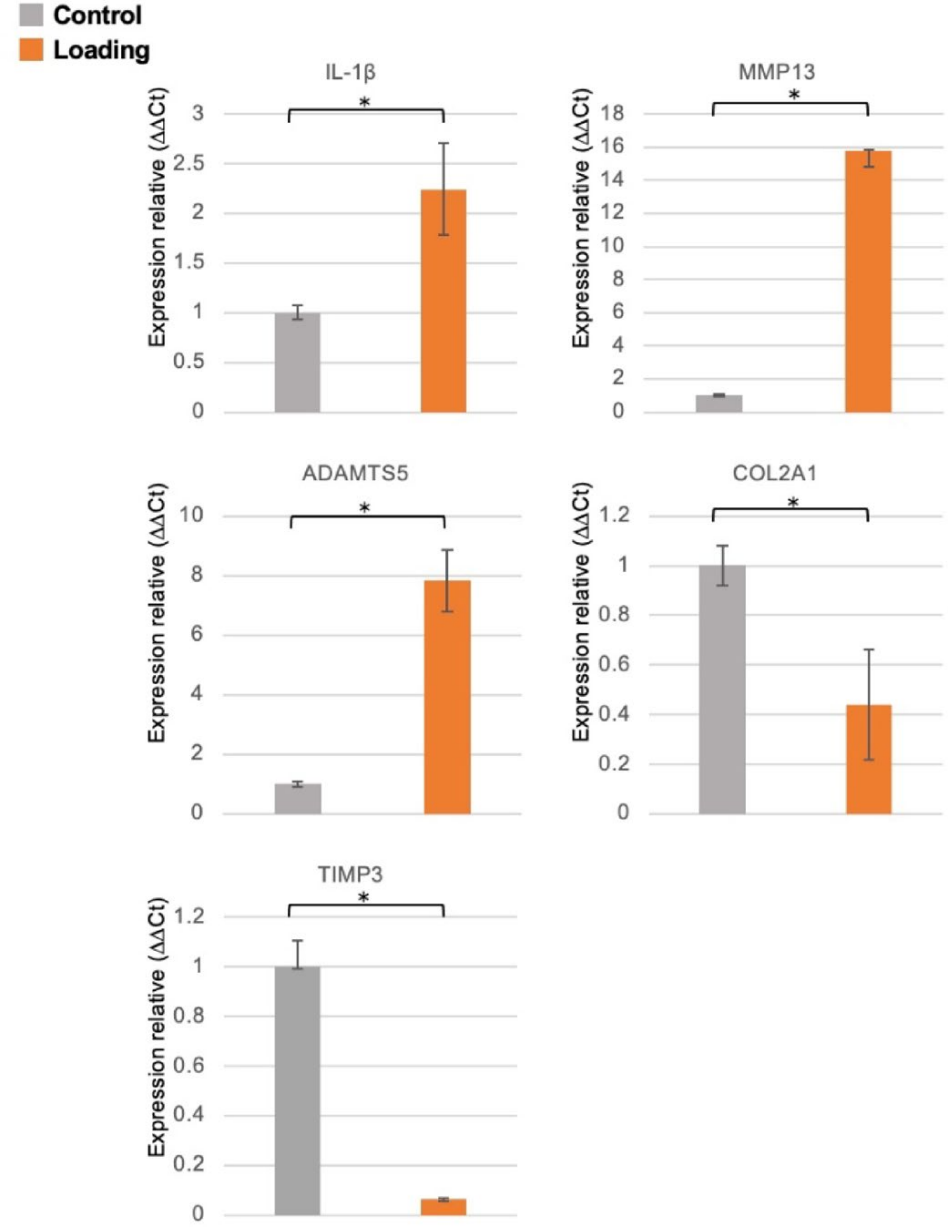
mRNA expression of inflammatory markers in mechanical loading (n = 6, mean ± SD, *p < 0.05). Gene expression was analyzed using the comparative Ct (ΔΔCt) method and normalized to GAPDH expression. IL-1β, MMP13, and ADAMTS5 mRNA expression was markedly increased, whereas COL2A1 and TIMP3 expression was decreased in the loading group compared to the control group.

The mRNA expression levels of MCC cells were characterized by quantitative reverse transcription PCR (qRT-PCR) analysis of collagen type I alpha 1 chain (COL1A1), collagen type II alpha 1 chain (COL2A1), collagen type III alpha 1 chain (COL3A1), aggrecan (ACAN), and SRY-box transcription factor 9 (SOX9), and were compared with those of rat skin fibroblasts. The results showed that the mRNA expression levels of COL2A1, ACAN, and SOX9 were significantly higher in MCC cells than in fibroblasts (*p* < 0.05), suggesting that the phenotypic expression of MCC cells is chondrogenic rather than fibroblastic (Fig. 1). As overloading of mandibular condyles increases the expression of interleukin (IL)-1 β, matrix metalloproteinases 13 (MMP13), and aggrecanase (ADAMTS5) and decreases the expression of tissue inhibitors of metalloproteinases (TIMP3) and COL2A1, this study examined whether the expression of these genes is altered in MCC cells under loading conditions using qRT-PCR analysis^18^. Under loading conditions, the IL-1β, MMP13, and ADAMTS5 mRNA expression levels increased, whereas TIMP3 and COL2A1 expression decreased, suggesting that MCC cells are reliable cells predominantly composed of chondrocytes and mixed with fibroblasts for this study (Fig. 2).

### Gene expression profiles

To comprehensively compare gene expression levels across groups, data analysis was performed using integrative Differential Expression and Pathway analysis (iDEP). After uploading the read count data to iDEP, iDEP correctly identified rats as the likely species based on the number of matching gene IDs. In total, 14,116 genes were identified after ID conversion and standard filtering. The log_2_(CPM + c) was selected for normalization between samples.

**Fig. 3:**
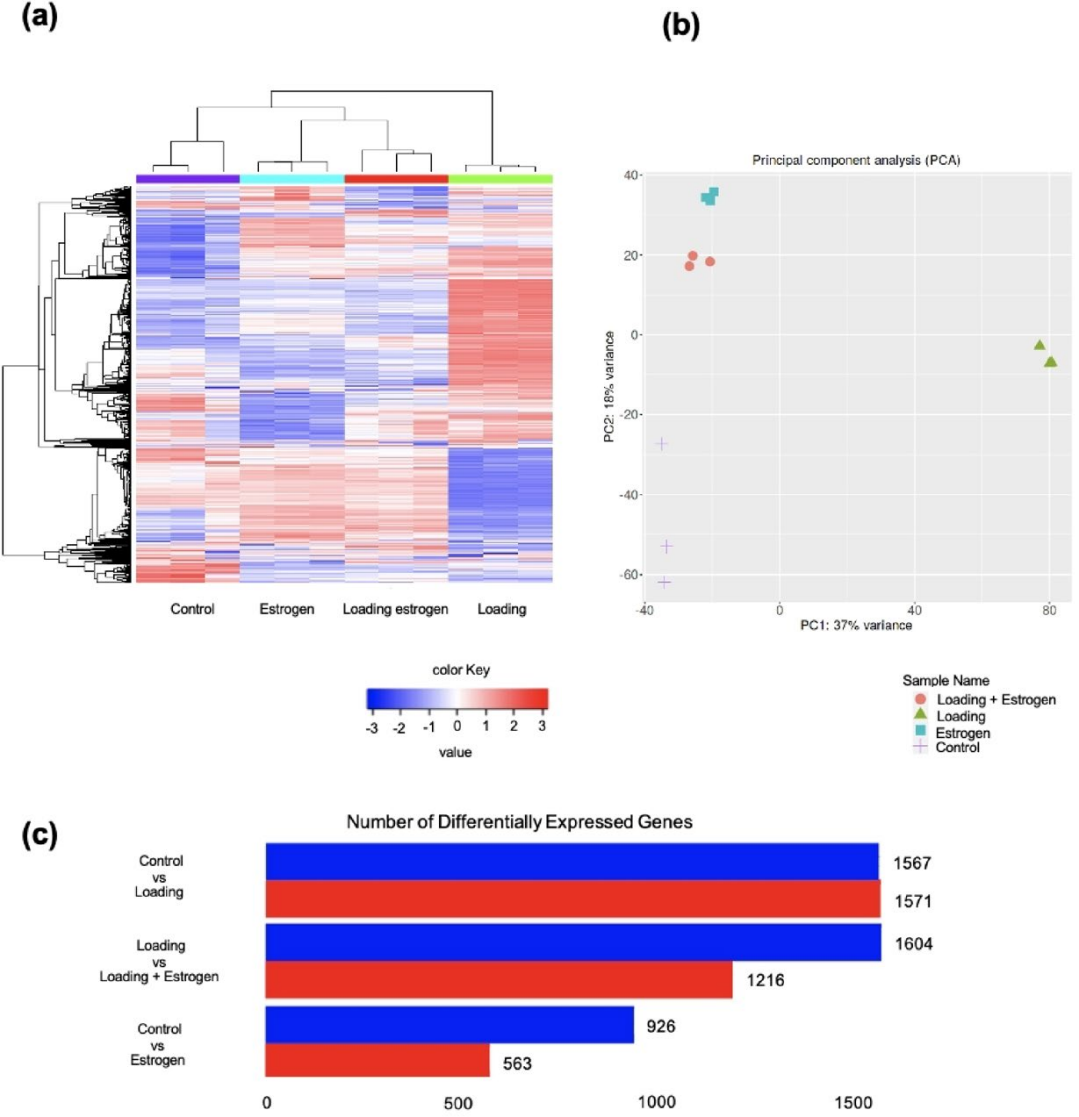
Gene Profiling. (**a**) Hierarchical clustering heat map and (**b**) principal component analysis showed clear changes in gene expression in the control, estrogen, and loading estrogen groups compared to the loading group. (**c**) Red indicates an increase, and blue indicates a decrease in differentially expressed genes (DEGs). Little difference existed in DEGs in the loading group relative to the control group, but approximately 400 genes were up- or downregulated in the loading estrogen group relative to the loading group and the estrogen group relative to the control group, respectively.

A hierarchical clustering heat map was created based on the top 1000 genes with the largest standard deviations (SD) of all samples (Fig. 3a). Comparisons of the loading, loading + estrogen, estrogen, and control groups showed differences in the aspect of their color mapping and gene expression. Principal component analysis (PCA) was performed to evaluate the similarities between the datasets (Fig. 3b). In addition to these hub genes, MMPs and ADAMTS have been reported to play a role in the activation of major degradative enzymes in articular cartilage, induction of chondrocyte apoptosis, ECM degradation, subchondral bone dysfunction, and synovial inflammation, ultimately leading to OA^19^. Therefore, we investigated the dynamics of gene expression. The gene expression of the substrate-degrading enzyme families MMP and ADAMTS observed in this study are shown (Table 1). Among the MMP family members, MMP2, MMP11, MMP13, MMP14, MMP15, MMP16, MMP19, and MMP23 were significantly upregulated, whereas MMP3, MMP9, and MMP10 were significantly downregulated in the loading group compared to the control group. MMP2, MMP3, MMP13, and MMP14 expression significantly increased, whereas MMP9, MMP23, MMP24, and MMP28 expression significantly decreased in the estrogen group compared to the control group. In the loading + estrogen group, relative to the control group, MMP3 and MMP10 levels were significantly increased, and MMP2, MMP9, MMP11, MMP13, MMP14, MMP15, MMP16, MMP19, MMP23, and MMP28 levels were significantly decreased. In contrast, among the ADAMTS family members, ADAMTS1, ADAMTS2, ADAMTS3, ADAMTS5, and ADAMTS6 were significantly upregulated, whereas ADAMTS8, ADAMTS14, ADAMTS15, and ADAMTS20 were significantly downregulated in the loading group compared to the control group. In the estrogen group, compared to the control group, ADAMTS1 and ADAMTS5 levels were significantly increased, whereas ADAMTSl4, ADAMTS6, ADAMTS8, ADAMTS9, ADAMTS10, ADAMTS14, ADAMTS17, and ADAMTS20 levels were significantly decreased. In the loading + estrogen group, relative to the control group, expression levels of ADAMTS1, ADAMTS8, ADAMTS10, ADAMTS14, and ADAMTS20 were significantly increased, and ADAMTS2, ADAMTSl2, ADAMTS3, ADAMTSl4, ADAMTS5, ADAMTS6, ADAMTS9, and ADAMTS17 were significantly decreased.

**Table 1:** Expression of MMP and ADAMTS genes in the extracellular matrix.

Gene	Control versus Loading		Loading versus Loading estrogen		Control versus Estrogen	
	Expression	*p*	Expression	*p*	Expression	*p*
MMP2	+	< 0.001	−	< 0.001	+	< 0.001
MMP3	−	< 0.001	+	< 0.001	+	< 0.001
MMP9	−	< 0.001	−	< 0.01	−	< 0.001
MMP10	−	< 0.01	+	< 0.01	+	
MMP11	+	< 0.01	−	< 0.001	−	
MMP13	+	< 0.001	−	< 0.001	+	< 0.001
MMP14	+	< 0.001	−	< 0.001	+	< 0.001
MMP15	+	< 0.001	−	< 0.01	+	
MMP16	+	< 0.001	−	< 0.001	+	
MMP17	−		+		−	
MMP19	+	< 0.001	−	< 0.001	−	
MMP23	+	< 0.001	−	< 0.001	−	< 0.05
MMP24	+		−		−	< 0.001
MMP28	−		−	< 0.001	−	< 0.001
ADAMTS1	+	< 0.001	+	< 0.001	+	< 0.001
ADAMTS2	+	< 0.001	−	< 0.001	−	
ADAMTSl2	+		−	< 0.05	−	
ADAMTS3	+	< 0.001	−	< 0.001	−	
ADAMTSl4	−		−	< 0.05	−	< 0.001
ADAMTS5	+	< 0.001	−	< 0.001	+	< 0.001
ADAMTSl5	+		−		−	
ADAMTS6	+	< 0.001	−	< 0.001	−	< 0.001
ADAMTS7	−		−		+	
ADAMTS8	−	< 0.001	+	< 0.001	−	< 0.001
ADAMTS9	−		−	< 0.001	−	< 0.05
ADAMTS10	+		+	< 0.01	−	< 0.05
ADAMTS14	−	< 0.05	+	< 0.05	−	< 0.05
ADAMTS15	−	< 0.05	+		+	
ADAMTS17	+		−	< 0.001	−	< 0.001
ADAMTS20	−	< 0.01	+	< 0.01	−	< 0.05

+ : gene expression upregulated, −: expression downregulated.

### Differentially expressed genes (DEGs)

DEG analysis was performed using DESeq, a false discovery rate (FDR) of 0.1, and a minimum magnification of 2 (Fig. 3c). Minor change was observed in gene expression in the loading group compared to that in the control group. However, approximately 400 genes were upregulated or downregulated in the estrogen group relative to the control group, the estrogen group relative to the control group, and the estrogen group compared to the control group.

### Gene ontology (GO) analysis

**Fig. 4:**
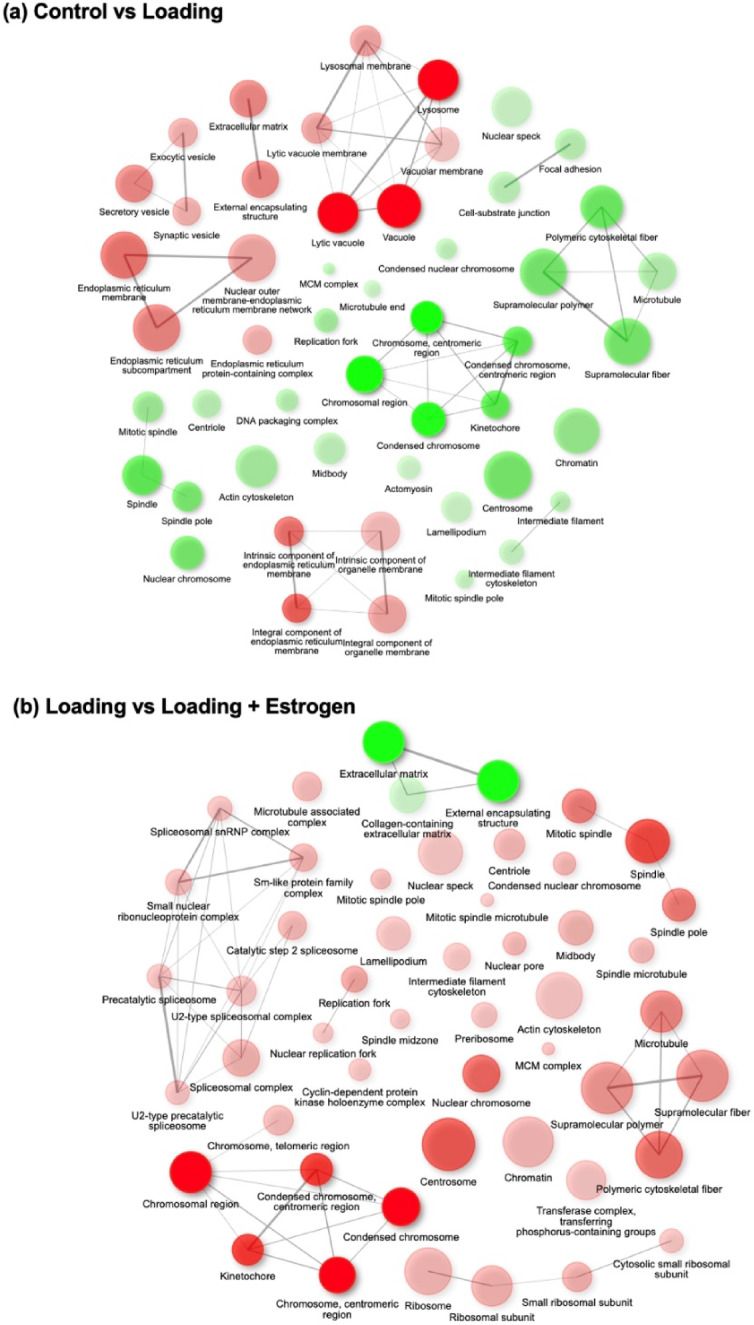

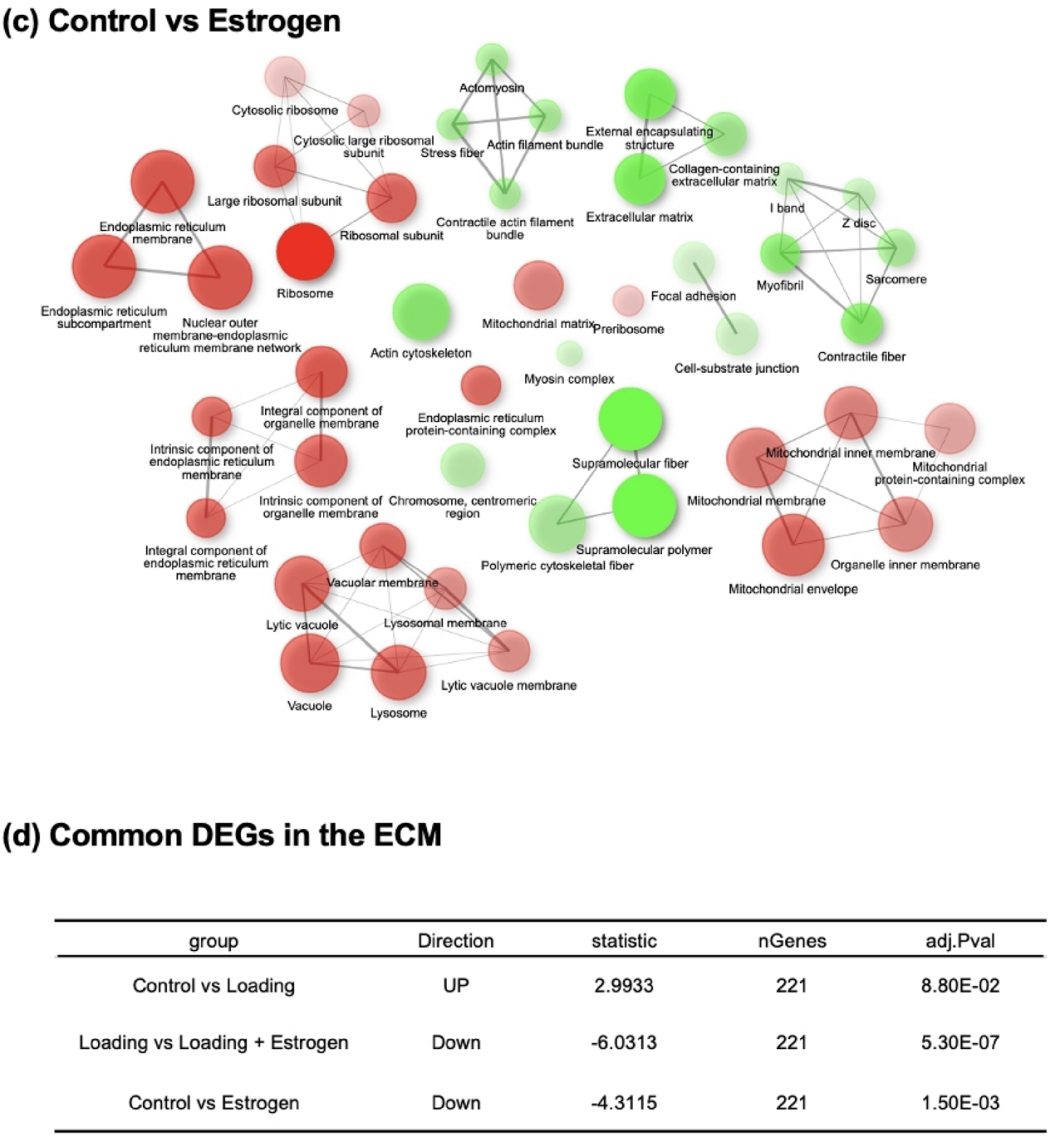
Gene Ontology analysis. The enrichment network of the top 50 GO terms, including both significantly upregulated and downregulated pathways, is shown. (**a**) Control group vs. Loading group: “External encapsulating structure” and “extracellular matrix” were increased in the loading group compared with the control group. (**b**) Loading group vs Loading + Estrogen group: “External encapsulating structure,” “extracellular matrix,” and “collagen-containing extracellular matrix” were decreased in the loading + estrogen group compared to the loading group. (**c**) Control group vs. estrogen group: The estrogen group showed decreased expression of “external encapsulating structure,” “extracellular matrix,” and “collagen-containing extracellular matrix” compared with the control group. (**d**) Common Differentially Expressed Genes (DEGs) in the ECM: Genes related to the extracellular matrix that were commonly differentially expressed across all comparisons. The circle color indicates the direction of enrichment (red: upregulated/enriched; green: downregulated/depleted). The intensity of the color reflects the magnitude of enrichment, with darker shades indicating stronger enrichment or depletion. The circle size corresponds to the number of genes associated with each GO term.

GO analysis of DEGs revealed the top 50 GO terms, including significantly increased and decreased GO terms (Fig. 4). The “external capsular structure” and “ECM” were increased in the loading group compared to the control group (Fig. 4a). However, a decrease in GO was observed in the estrogen group compared to that in the control group (Fig. 4b, c). GO analysis of the top 50 pathways revealed that “ECM” and “external encapsulating structures” were commonly observed across all comparisons among the three groups. The term “external encapsulating structure” generally refers to the outer layer surrounding and supporting cells or entire organisms, primarily non-animal organisms such as plants, fungi and bacteria. However, in the context of this study, “external encapsulating structure” may be considered synonymous with the “extracellular matrix (ECM)”, which plays a critical role in supporting the structure and function of mammalian cells. Therefore, in this study, we focused on the ECM. GO analysis identified 221 common DEGs related to the ECM in each group (Fig. 4d).

### Protein–protein interaction analysis (PPI analysis)

**Fig. 5:**
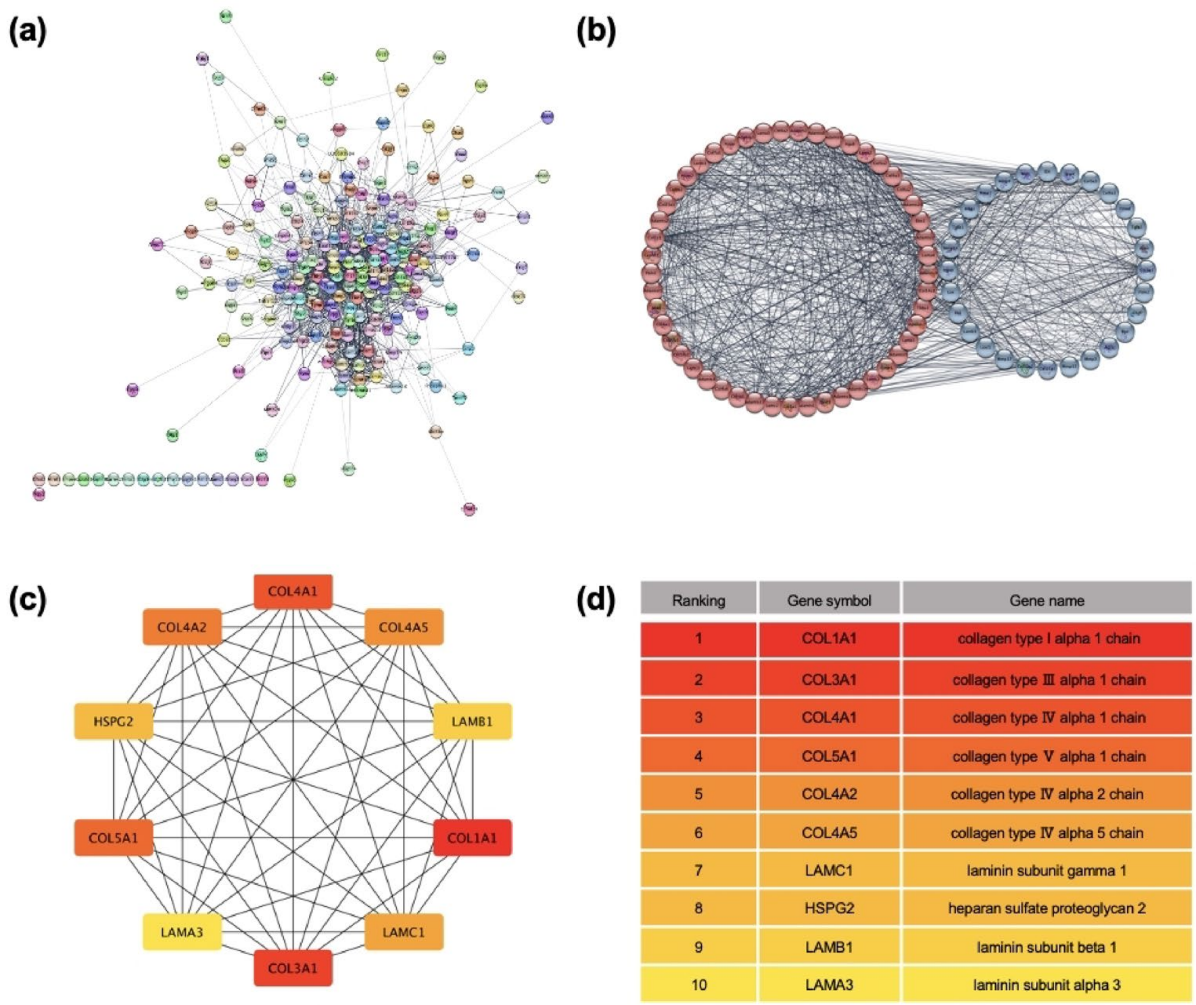
PPI analysis and hub genes and PPI networks. (**a**) PPI network constructed using the STRING online PPI construction tool. (**b**) MCODE clusters were used to evaluate the importance of the clustered network in the extracellular matrix selected by clustering scores, and the top two clusters with the highest clustering scores were used for screening to identify 77 genes. (**c**, **d**) Top 10 STRING networks ranked using the MCC method. The 10 hub genes were identified: COL1A1, COL3A1, COL4A1, COL5A1, COL4A2, COL4A5, LAMC1, HSPG2, LAMB1, and LAMA3 were identified.

PPI analysis was performed to investigate the interrelationships among the 221 genes associated with the “ECM”, which showed changes according to the GO analysis. A PPI network was constructed by selecting genes with a composite score of 0.4 using the STRING online PPI construction tool (Fig. 5a). MCODE cluster analysis, which groups proteins with strong interrelationships, was performed using Cytocluster (node score cutoff = 0.2, order cutoff = 2, K-core = 2, maximum depth value = 100). Seven clusters were formed, and the most significant clustering scores were observed in the primary MCODE cluster. The top two clusters with the highest clustering scores were selected to screen and identify the hub genes (Fig. 5b). The top 10 genes ranked using the MCC method were COL1A1, COL3A1, COL4A1, COL5A1, COL4A2, COL4A5, LAMC1, HSPG2, LAMB1, and LAMA3 (Fig. 5c, d).

### Verification of hub genes using qRT-PCR analysis

**Fig. 6:**
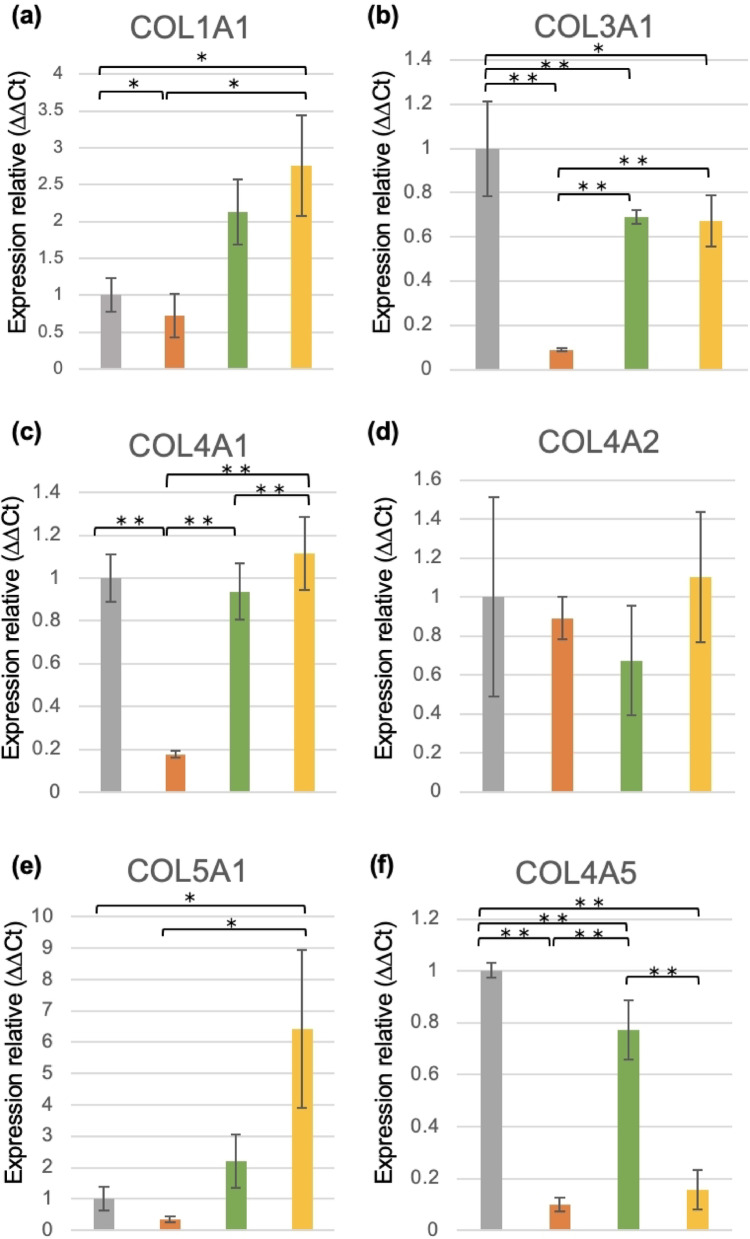

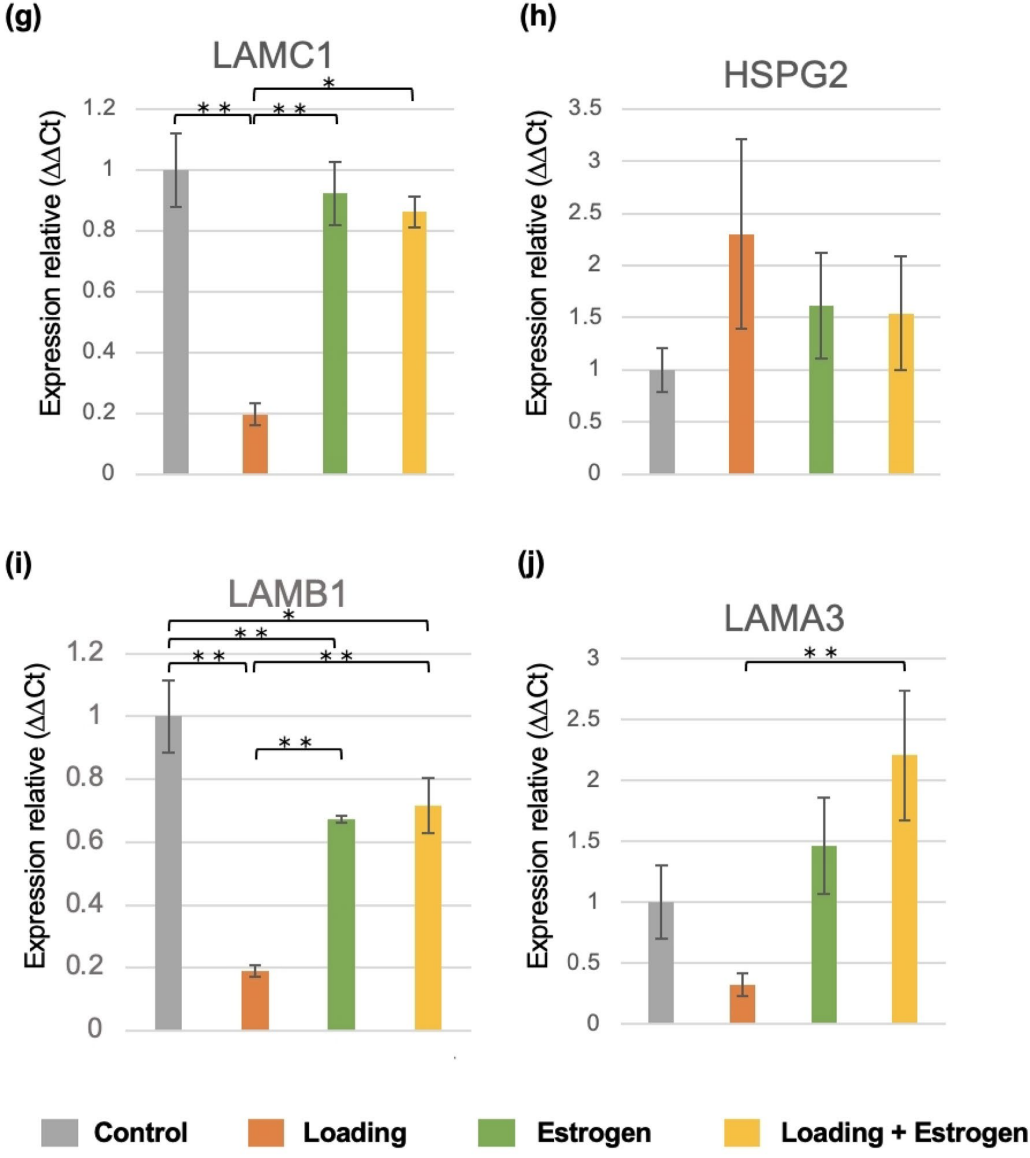
Gene expression in each hub gene. (**a**, **b**, **c**, **d**, **e**, **f**, **g**, **i**, and **j**) Expression of COL1A1, COL3A1, COL4A1, COL4A2, COL5A1, COL4A5, LAMC1, LAMB1, and LAMA3 was decreased in the loading group relative to the control group and increased in the loading + estrogen group relative to the control group (n = 6, mean ± SD, *p < 0.05, ** p < 0.01). (**b**, **c**, **d**, **f**, **g**, and **i**) In addition, the expression of COL3A1, COL4A1, COL4A2, COL4A5, LAMC1, and LAMB1 decreased in the estrogen group compared to that in the control group. Gene expression was analyzed using the comparative Ct (ΔΔCt) method and normalized to GAPDH expression.

qRT-PCR analysis was performed to examine the changes in mRNA expression of the hub genes identified through PPI analysis within each group. qRT-PCR analysis showed that COL3A1, COL4A1, COL4A2, COL4A5, LAMC1, and LAMB1 mRNA expression levels decreased in the control and estrogen-treated groups. This decrease was more pronounced in the loading group than in the control group. In contrast, COL1A1, COL3A1, COL4A1, COL4A2, COL5A1, COL4A5, LAMC1, LAMB1, and LAMA3 mRNA expression levels increased in the loading + estrogen group (Fig. 6).

### Western blotting analysis

**Fig. 7:**
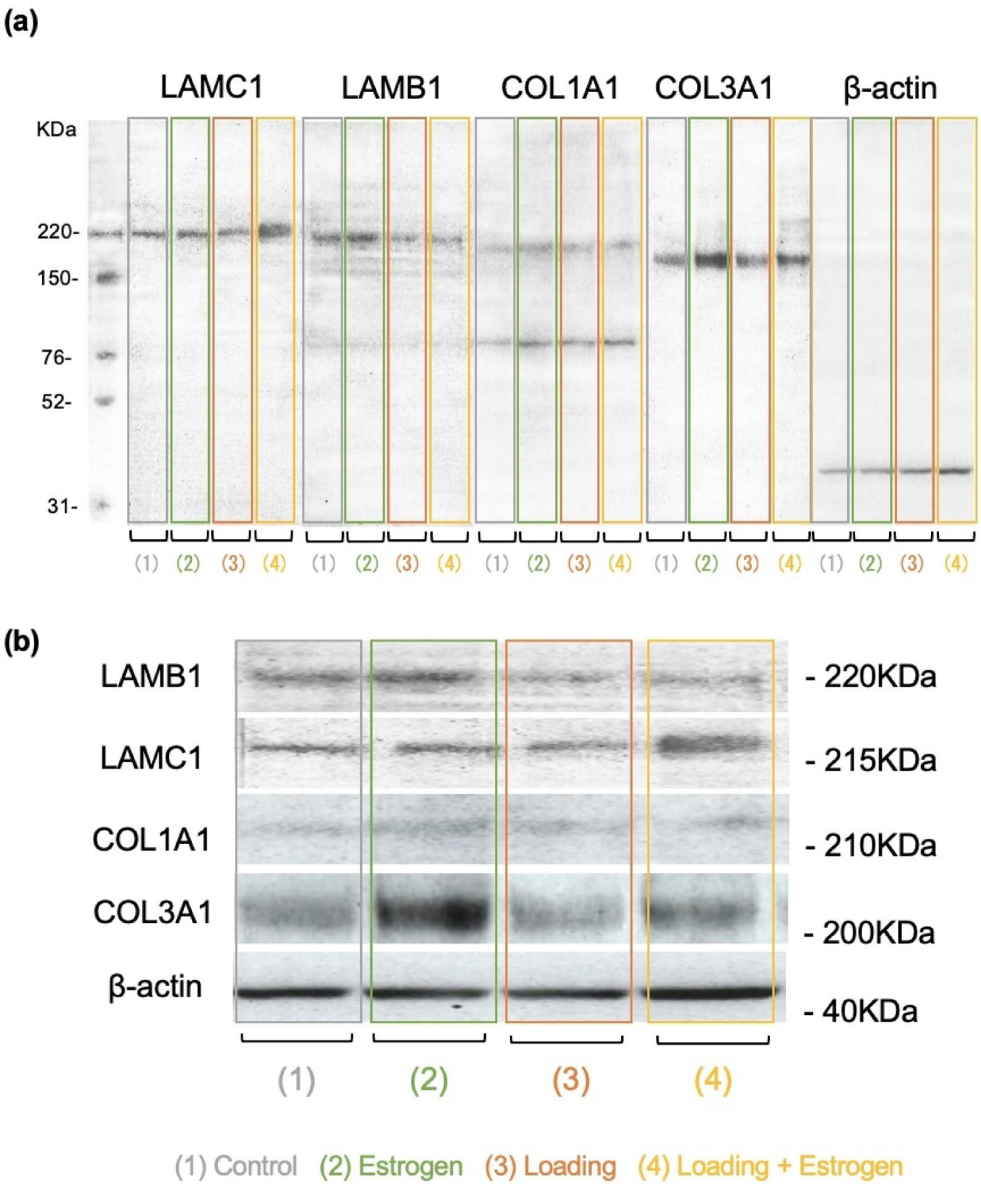

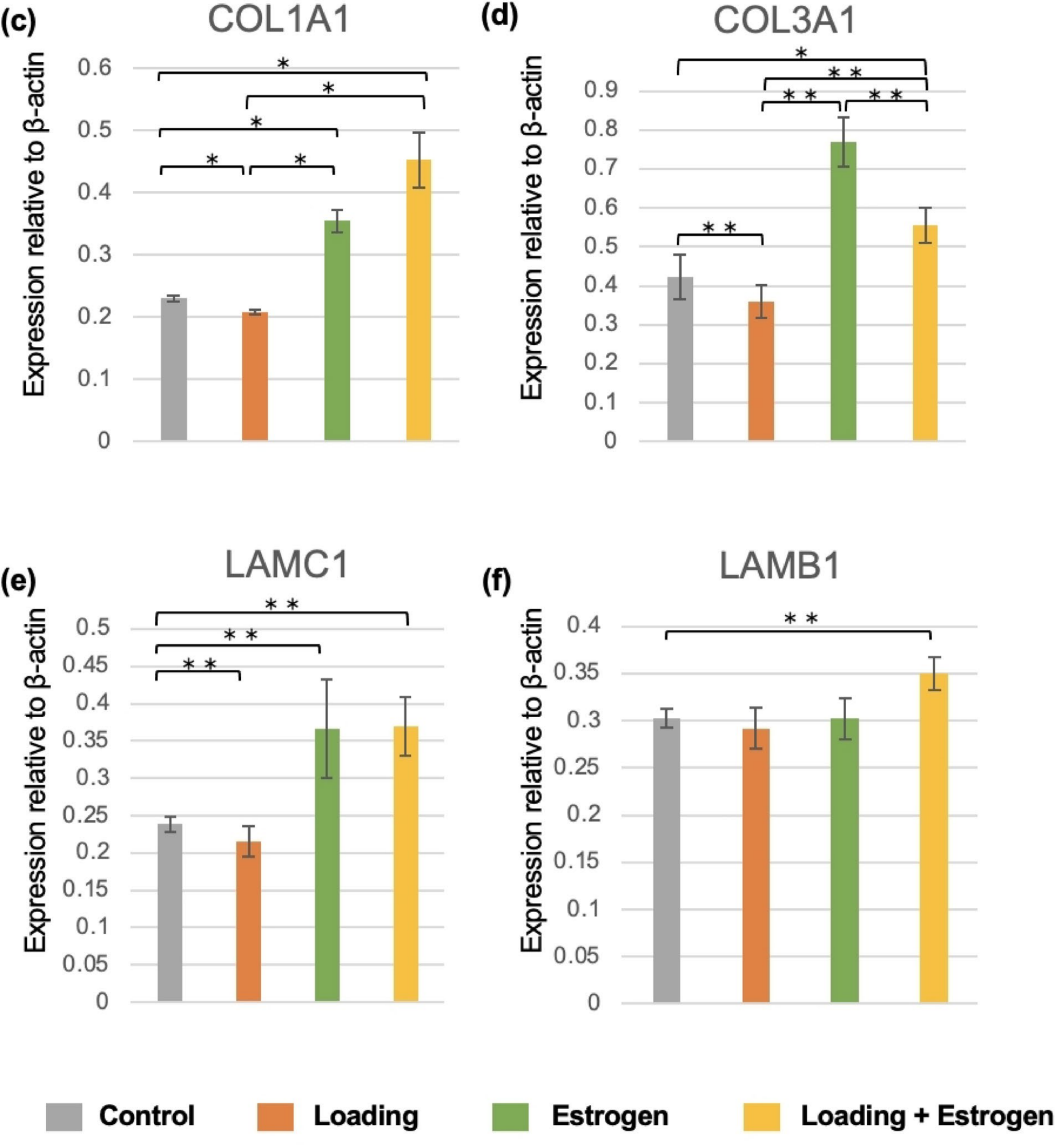
Protein expression in each hub gene. (**a**, **b**) Western blotting results are shown. (**c**, **d**, **e**, and **f**) Protein expression of COL1A1, COL3A1, LAMC1, LAMB1, and LAMA3 was decreased in the loading group compared to the control group and increased in the loading + estrogen group compared to the control group. (**c**, **f**) Protein expression of COL1A1 and LAMB1 was decreased in the estrogen group compared to the control group. (n = 6, mean ± SD, *p < 0.05, **p < 0.01).

The protein expression levels of COL1A1, COL3A1, LAMC1, and LAMB1, which showed significant differences in the qRT-PCR analysis, were investigated. COL1A1, COL3A1, and LAMC1 protein expression levels were significantly lower in the loading group than in the control group. The protein expression levels of COL1A1 and COL3A1 were significantly higher in the loading + estrogen group than in the loading group (Fig. 7).

## Discussion

To the best of our knowledge, this study is the first to observe the effects of estrogen and mechanical loading on MCC cells to elucidate the pathogenesis of TMJ-OA at the gene level. TMJ-OA progression involves damage to the superficial fibrous layer, which is directly subjected to loading and gradually extends to the deeper cartilage layers^13^. Therefore, changes in the superficial layer of the mandibular condylar cartilage may contribute to the onset of OA. In this study, we cultured cells from the condylar cartilage, which is predominantly composed of chondrocytes mixed with fibroblasts, to investigate the effects of estrogen on mechanically loaded group cells. GO analysis revealed that ECM-related genes were depleted in all estrogen-treated groups. Therefore, we focused on ECM-related genes using genome-wide analysis data. We observed various alterations in ECM-related genes under loading, estrogen, and loading combined with estrogen treatment. At the protein level, COL1A1 and COL3A1 decreased under loading conditions but increased under loading combined with estrogen conditions. Estrogen may be, at least in part, significantly involved in TMJ-OA via COL1A1 and COL3A1; however, the mechanism by which these proteins affect TMJ-OA remains unclear.

We conducted a detailed exploration of related molecules at the gene expression level. GO analysis of the top 50 pathways revealed that the ECM and external encapsulating structure were commonly observed across all comparisons among the three groups. In this study, we focused on the ECM. MCODE cluster analysis of the 221 ECM genes yielded seven clusters. The top two clusters were selected to screen and identify hub genes. A total of 77 genes were identified in the top two clusters, and 10 hub genes were ultimately detected using the MCC method in CytoHubba. These genes included COL1A1, COL3A1, COL4A1, COL5A1, COL4A2, COL4A5, LAMC1, HSPG2, LAMB1, and LAMA3. COL1A1, COL3A1, and COL5A1 are primarily observed in fibroblasts^20,21^, whereas other genes are predominantly expressed in chondrocytes^22^. Almost no expression of type IV collagen or laminin is observed in the fibrous layer of the mandibular condyle^23,24^. Therefore, the expression levels of type I, III, and V collagens are thought to originate from fibroblasts, whereas the levels of type II and IV collagens, laminin, and HSPG2 probably originate from chondrocytes.

In addition to these hub genes, MMPs and ADAMTS have been reported to play a role in the activation of major degradative enzymes in articular cartilage, induction of chondrocyte apoptosis, ECM degradation, subchondral bone dysfunction, and synovial inflammation, ultimately leading to OA. Therefore, we investigated the dynamics of these genes (Table 1). Increased MMP2, MMP13, MMP14, and ADAMTS5 expression was observed under loading and estrogen conditions. However, their expression decreased under combined loading and estrogen conditions in this study. MMPs are key players in ECM degradation. Among them, MMP13 cleaves collagen types I, II, and III and targets various ECM components^25^. ADAMTS5 plays a pivotal role in cartilage breakdown by degrading aggrecan, a major component of cartilage^26^. Type II collagen, another essential structural component of cartilage, is primarily degraded by matrix metalloproteinases, particularly MMP13 (collagenase 3)^25^.

Although ADAMTS5 and MMP13 contribute to cartilage damage, they act on different ECM targets. ADAMTS5 degrades aggrecan by exposing type II collagen, which is more susceptible to degradation by MMPs^27^. The balance between the increased and decreased expression of these enzymes likely determines whether ECM destruction or protection takes place. Among the 10 hub ECM-related genes identified by CytoHubba, six and three showed decreased expression under loading and estrogen conditions, respectively, whereas seven were upregulated under combined loading and estrogen conditions. These patterns were consistent with the altered expression of MMP2, MMP13, MMP14, and ADAMTS5 observed in this study.

An exception to these findings was the increased COL1A1 protein expression in the estrogen group, despite the upregulation of MMPs and ADAMTS5. Previous studies have reported that estrogen stimulates type I collagen production via Transforming Growth Factor-β (TGF-β) signaling in dermal fibroblasts^28^. Elevated COL1A1 production may not be sufficient to counteract degradation mediated by MMPs and ADAMTS5. However, further studies are required to elucidate this phenomenon.

Decreased expression of ADAMTS8, ADAMTS14, and ADAMTS20 was observed under loading and estrogen conditions, whereas their expression increased under the combined loading and estrogen conditions. ADAMTS8 is primarily known for its antiangiogenic properties, which help maintain the avascular nature of cartilage^29^. ADAMTS14 plays a key role in procollagen processing and maturation and stabilizes the ECM^30^. ADAMTS20 is involved in proteoglycan metabolism, including aggrecan degradation, and regulates the cartilage ECM^31^. Reduced expression of these ADAMTS enzymes under loading or estrogenic conditions may lead to vascularization, ECM destabilization, and dysregulation of cartilage homeostasis.

Overall, loading and estrogen conditions individually contributed to cartilage damage via ECM degradation. However, their combined application promotes cartilage protection and repair by stimulating ECM synthesis. The apparent contradiction that loading or estrogen alone promotes degradation, whereas their combination exerts protective effects, can be explained by the dual nature of estrogen and its condition-dependent interactions with mechanical loading. Estrogen receptor-α plays a critical role in chondrocyte mechanotransduction, where mechanical stimuli modulate the effects of estrogen on ECM remodeling^32^. Estrogen treatment has been shown to improve the balance between TIMPs and MMPs, suggesting that it can modulate ECM remodeling depending on physiological conditions^33^.

Altered TIMP expression may explain this phenomenon. TIMP3, which inhibits both MMPs and ADAMTS enzymes, showed decreased expression under loading or estrogenic conditions, whereas no significant changes were observed under combined loading and estrogen conditions. This reduction in TIMP3 levels may contribute to the degradation observed with loading or estrogen alone, whereas stable expression under combined conditions may mediate ECM protection. Further investigation is necessary to clarify how estrogen modulates ECM degradation under such loading conditions. The results are summarized in Supplementary Fig. 1.

In conclusion, this study demonstrated the effects of estrogen on mechanical loading using MCC cells to elucidate the mechanisms underlying TMJ. Estrogen may be, at least in part, significantly involved in TMJ-OA via altering of the ECM levels such as collagen, laminin, MMPs, and ADAMTS.

## Materials and methods

### Preparation of primary MCC cells

All animal experiments were conducted with the approval of the Animal Care and Use Committee of the Health Sciences University of Hokkaido (Approval No. 22-019), and the experimental protocol was reported in accordance with the ARRIVE guidelines.

Three-8-week-old female Wistar rats (180–200 g; Sankyo Laboratories, Osaka, Japan) were used in the experiment and housed in the Animal Experiment Center of the Health Sciences University of Hokkaido. They were kept in cages under a 12 h light/dark cycle at controlled temperatures (23 ± 2 °C) with free access to standard rat chow and water.

For mandible sample collection, the rats were deeply anesthetized with 2% isoflurane (Forrane®) inhalation and euthanized by gradual exposure to carbon dioxide (CO_2_) at a displacement rate of 20–30% of the chamber volume per minute, followed by cervical dislocation to confirm death.

MCC cells were then isolated from the mandible. The tissue on the surface of the mandible was detached using a stereomicroscope after excising the surrounding tissues. Cells were isolated from detached tissues using the outgrowth method^34^. Tissue slices were placed on type I collagen-coated culture dishes (IWAKI, Shizuoka, Japan), and 3.0 mL of Dulbecco’s modified Eagle’s medium (DMEM; Sigma-Aldrich, MO, USA) was added to the culture dishes. When the cells reached 70–80% confluence, they were detached using 0.25% trypsin/0.02% EDTA (Thermo Fisher Scientific, MA, USA). Early passage cells (primary to fifth generation) were used in all experiments. As phenol red has estrogen-like activity, phenol red-free DMEM was used in the experiment.

### Cell culture

MCC cells were maintained in DMEM supplemented with 10% (v/v) fetal bovine serum (Thermo Fisher Scientific) and 5% (v/v) penicillin–streptomycin (Thermo Fisher Scientific) in a 37 °C, 5% CO_2_ humidified incubator (Sanyo) at 37 °C, 5% CO_2_” has been changed to “in a humidified incubator (Sanyo) set at 37 °C with 5% CO_2_. In all experiments, cells were seeded on 100 mm type I collagen-coated culture dishes (IWAKI) and allowed to grow until 70% confluence. The adult rat skin fibroblast cell line was obtained from TOYOBO Co., Ltd. (Osaka, Japan). The cells were thawed under sterile conditions after cryopreservation. They were then cultured in Rat Fibroblast Growth Medium (Cell Applications), supplemented with the recommended growth supplements, at 37 °C in a humidified atmosphere with 5% CO_2_ until reaching approximately 70% confluency.

### Application of mechanical loading and addition of estrogen (17β − estradiol)

Mechanical loading of MCC cells was performed using the compression method described by Yamaguchi et al.^35^. In a preliminary experiment, a continuous compression force of 10.0 g/cm^2^ was applied to the MCC cells for 3 h (Supplementary Fig. 2). Primer sequences used in this study are listed in Supplementary Table 1. The cultured MCC cells were harvested and analyzed using qRT-PCR to examine the mRNA expression of inflammatory markers IL-1β, MMP13, and ADAMTS5; the proteolytic enzyme TIMP3; and COL2A1, a specific marker of chondrocytes. In the control group, glass plates without weights were placed on the cells. To determine the estrogen concentration used in this study, we referred to a previous report^36^ and selected a physiologically high concentration of 10⁻⁶ mol/L, which was added to the culture medium. A stock solution of estrogen dissolved in ethanol (2.5 mM) was prepared and added to the medium to achieve a final estrogen concentration of 10^−6^ mol/L (1.0 µM) and an ethanol concentration of ≤ 0.1%.

### RNA sequencing and bioinformatics methods

Cultured mandibular head surface layer cells from each group were collected after 3 h, and total RNA was extracted using an RNeasy Mini Kit (Qiagen, Hilden, Germany). A BioAnalyzer (Agilent 2100; Agilent, Santa Clara, CA, USA) was used to assess the integrity of the total RNA samples. Only high-quality RNA samples (RIN ≥ 9.0) were used to construct the sequencing library. Using a template prepared by the strand-specific library preparation method (dUTP method), PCR amplification was performed with a primer controlling the index sequence to prepare a sequence library (NEBNext Poly(A) mRNA Magnetic Isolation Module; NEBNext Ultra II Directional RNA Library Prep Kit for Illumina). RNA-Seq data were obtained using a NovaSeq 6000 system (Illumina, San Diego, CA, USA). Sequence reads were trimmed using Trimmomatic (ver. 0.38). Trimmed sequence reads were mapped to the reference genome (Rnor 6.0) using HISAT2 (ver. 2.1.0). The raw read count for each gene was calculated using FeatureCounts (ver. 1.6.3). The raw read counts were uploaded to iDEP.96 (http://bioinformatics.sdstate.edu/idep96/) for hierarchical clustering, principal component analysis (PCA), correlation analysis, heatmap generation, and functional enrichment analysis. The initial settings of the iDEP were used for the analysis.

### Quantitative reverse transcriptase PCR

The extracted RNA was reverse transcribed to cDNA using Omniscript Reverse Transcriptase (Qiagen, Hilden, Germany). The mRNA expression levels were measured using a StepOne Real-Time PCR System (Thermo Fisher Scientific). β-actin (used as an internal standard) and four target genes—IL-1β, MMP13, ADAMTS5, TIMP3, and COL2A1—were identified using the MCC method to assess the gene expression levels in response to the loading model. Primers were designed for 10 genes: collagen type I alpha 1 chain (COL1A1), collagen type III alpha 1 chain (COL3A1), collagen type IV alpha 1 chain (COL4A1), collagen type V alpha 1 chain (COL5A1), collagen type IV alpha 2 chain (COL4A2), collagen type IV alpha 5 chain (COL4A5), laminin subunit gamma 1 (LAMC1), heparan sulfate proteoglycan 2 (HSPG), laminin subunit beta 1 (LAMB1), and laminin subunit alpha 3 (LAMA3) (Fig. 5c, d). Real-time PCR was performed using the obtained cDNA, QuantiTect SYBR Green PCR kit (Qiagen), and a pair of primers. The PCR conditions included initial pre-incubation at 95 °C for 3 min, denaturation at 95 °C, 40 cycles of denaturation at 95 °C for 10 s, annealing at 60 °C for 20 s, and elongation at 72 °C for 1 s. The relative expression levels of each mRNA were calculated as Ct (value obtained by subtracting the Ct value of GAPDH mRNA from the Ct value of target mRNA) using the ∆∆Ct method. The amount of target mRNA relative to GAPDH mRNA was expressed as 2^−(∆Ct)^.

### Western blot analysis

MCC cells were washed twice with phosphate-buffered saline and lysed in RIPA buffer (ATTO, Tokyo, Japan) containing protease inhibitors. Cellular protein concentrations were determined using a BCA protein assay kit (Thermo Fisher Scientific). Cell lysates were mixed with sodium dodecyl sulfate (SDS) sample loading buffer and boiled at 100 °C for 10 min. Proteins were separated using SDS − PAGE on a 10% polyacrylamide gel. After electrophoretic separation, the proteins were transferred to a 0.22 mm polyvinylidene fluoride membrane. The blot membranes were blocked in 5% bovine serum albumin for 2 h at room temperature and incubated with primary antibodies overnight at 4 °C. The secondary antibody used was goat anti-mouse IgG (Thermo Fisher Scientific). The samples were then washed with phosphate-buffered saline with Tween 20. Proteins were detected using enhanced chemiluminescence detection reagents (Thermo Fisher Scientific), and the signals were normalized to β-actin. Images were analyzed using ImageJ software (National Institutes of Health, Bethesda, MD, USA). The primary antibodies used in this study include (1:1000, sc-17751) and LAMB1 (1:1000, sc-17810) from Santa Cruz Biotechnology and β-actin (1:1000, A2228, Sigma-Aldrich).

### Statistical analysis

Statistical analyses were performed using SPSS version 29 software (SPSS, Inc., Chicago, IL, USA). The results were compared using an unpaired t-test for two-group comparisons and one-way ANOVA followed by Tukey’s post hoc test for multiple comparisons. Data are expressed as mean ± SD. Data are expressed as mean ± SD, with *p* values < 0.05 indicating statistical significance.

## Supplementary Information

Supplementary Information.

## Data Availability

The datasets generated and/or analyzed during the current study are available in the NCBI Sequence Read Archive (SRA) repository, accession no. SRP564479 (https://www.ncbi.nlm.nih.gov/sra?term=SRP564479). The datasets generated and/or analyzed during the current study are available from the corresponding author upon reasonable request. For data requests related to this study, please contact the corresponding author.
