# Supplementary material for: Effects of estrogen and mechanical loading on cultured cells derived from mandibular condylar cartilage
Source: Sci Rep. 2025 Jul 2;15:23470. doi: 10.1038/s41598-025-07770-4 (PMC12223048; doi:10.1038/s41598-025-07770-4)
Supplement: Supplementary file 1 — Supplementary Information. [file 41598_2025_7770_MOESM1_ESM.pdf]

Supplementary Information

Effects of estrogen and mechanical loading on cultured cells derived from mandibular condylar cartilage

Jin Tsuchida[1], Yuya Nakao[1,*], Koken Sato[1], Osamu Uehara[2], Itaru Mizoguchi[3], Yoshihiro Abiko[4], Masahiro Iijima[1]

[1]Division of Orthodontics and Dentofacial Orthopedics, Department of Oral Growth and Development, School of Dentistry, Health Sciences University of Hokkaido, 1757 Kanazawa, Ishikari−Tobetsu, Hokkaido 061−0293

[2]Division of Disease Control and Molecular Epidemiology, Department of Oral Growth and Development, School of Dentistry, Health Sciences University of Hokkaido, 1757 Kanazawa, Ishikari−Tobetsu, Hokkaido 061−0293

[3]Division of Orthodontics and Dentofacial Orthopedics, Tohoku University Graduate School of Dentistry, 4-1 Seiryo-machi, Aoba-ku, Sendai 980-8575, Japan

[4]Division of Oral Medicine and Pathology, Department of Human Biology and Pathophysiology, School of Dentistry, Health Sciences University of Hokkaido, 1757 Kanazawa, Ishikari−Tobetsu, Hokkaido 061−0293, Japan

*Correspondence: Yuya Nakao
E-mail : y-nakao@hoku-iryo-u.ac.jp

Supplementary Figure 1. Schematic diagram of the key findings of this study

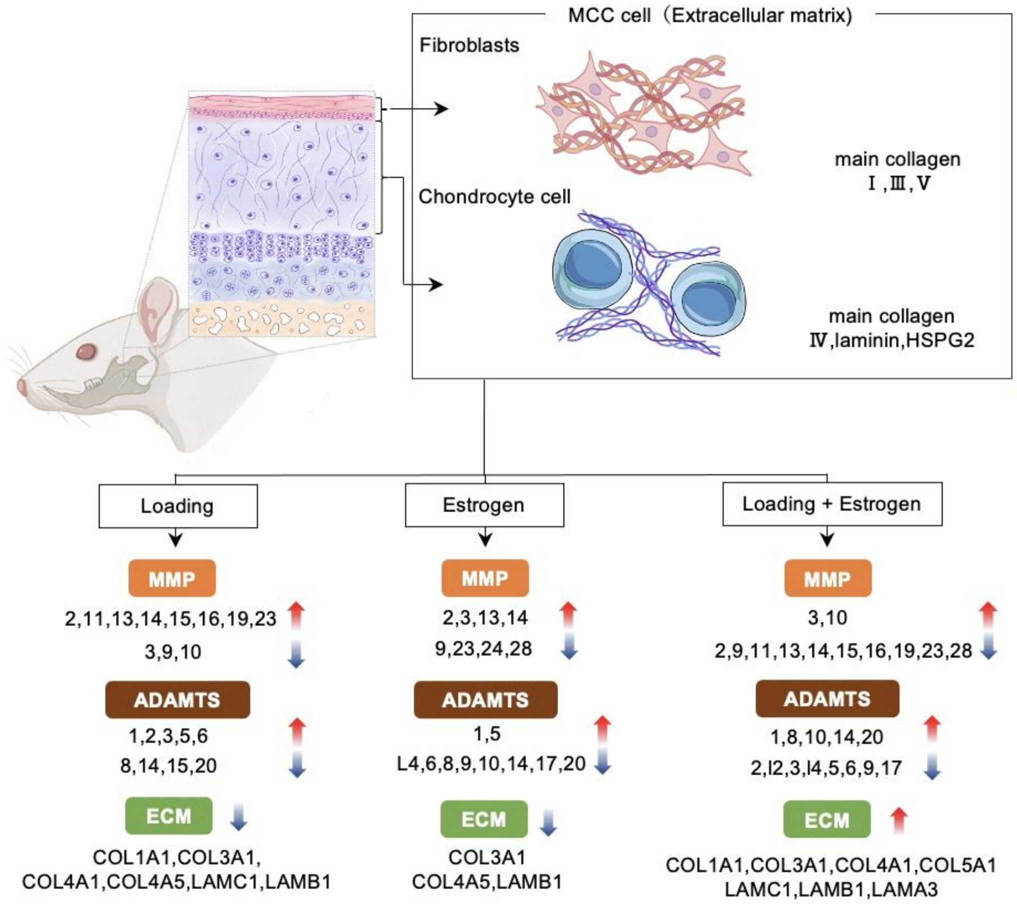

Supplementary Figure 2. Mechanical loading and estrogen (E) addition in cultured mandibular head surface cells.

(a) MCC cells were grown to confluency. A cover glass was placed over the cultured cells, and a 10 g/cm^2 weight was placed on the cover glass. Estrogen was added to the culture medium before the cover glass was placed. In the control group, only a cover glass was placed. (b) loading estrogen group, (c) loading group, (d) estrogen group, and (e) control group (n = 3).

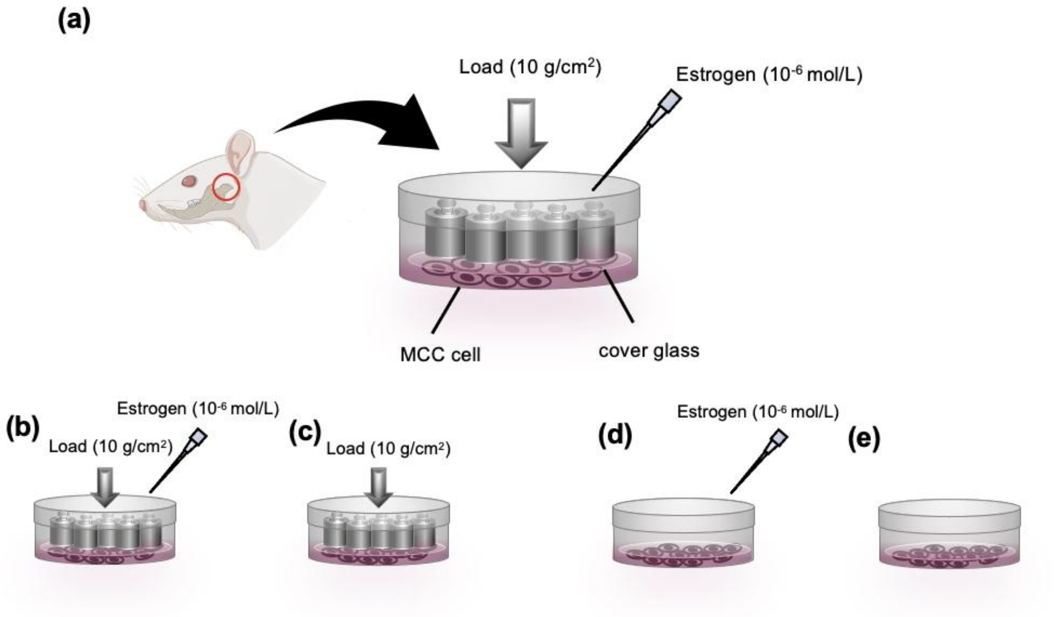

Supplementary Figure 3. Uncropped western blots

Original, uncropped films showing (a) LAMB1, (b) LAMC1, (c) COL1A1, (d) COL3A1 and (e) β-actin. In panels (a, b and c), the HiMark Pre-Stained Protein Standard™ (31–460 kDa) was loaded at both ends of each gel to indicate molecular weight. In panels (a, b, c, d and e), samples are arranged from left to right as control, estrogen, loading and loading + estrogen groups. Panels (a, d and e) correspond to lanes 1–4; panel (c) to lanes 5–8; and panel (b) to lanes 9–12.

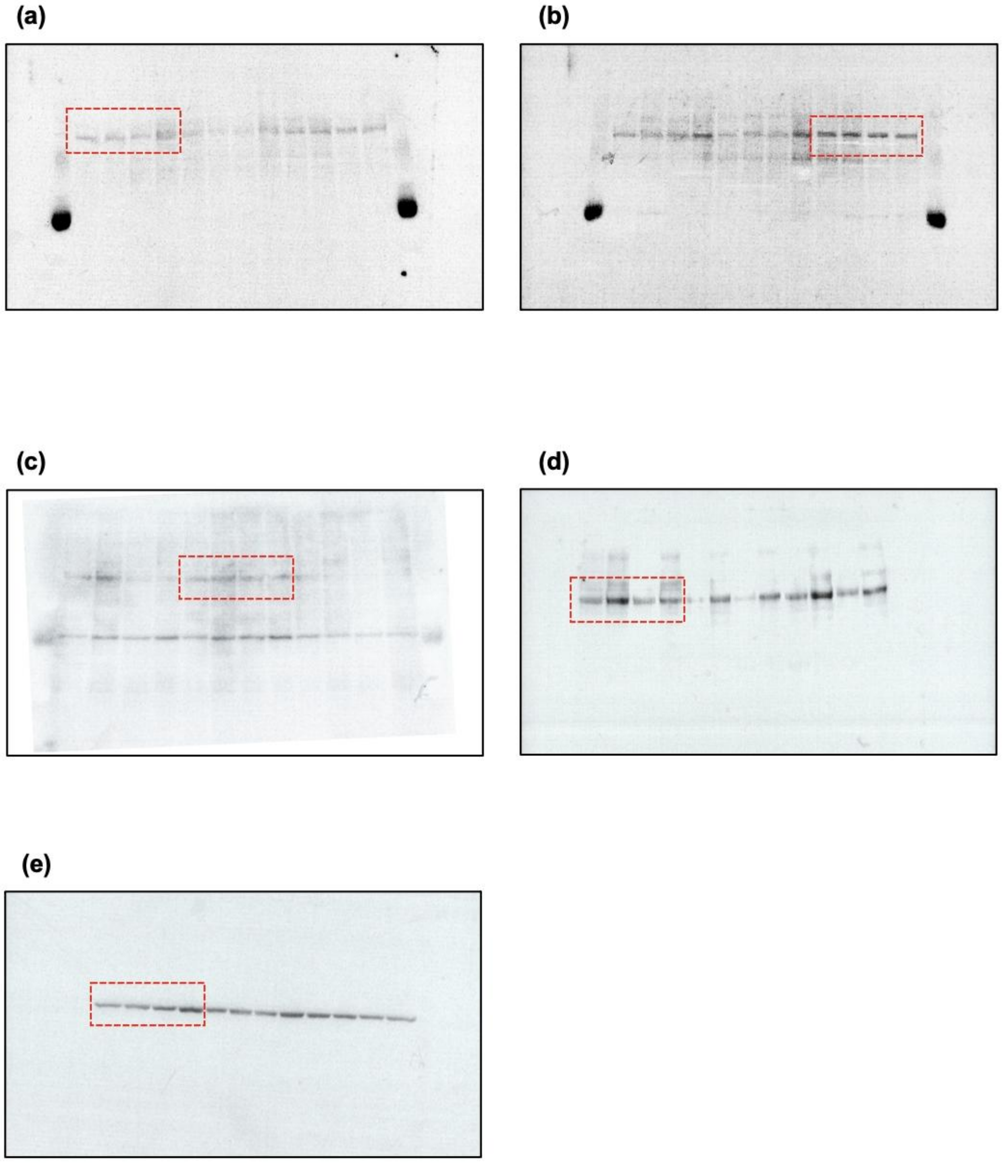

Supplementary Table 1. Primers for qRT-PCR Analysis

Molecule	sense	primer sence	Amplicon length	Accession no.
COL1A1	Sence(5'-3')	AACCTGGATGCCATCAAGG	142	XM_032912698.1
	Antisense(5'-3')	ATGACCGATGGATTCCAGTT		
COL2A1	Sence(5'-3')	TCCTGCTGGAAAAGATGGTC	62	NM_012929.1
	Antisense(5'-3')	GTGACCCTGGACTTCAAGGA		
COL3A1	Sence(5'-3')	TCCTGATCAAGAATTTGGTGTG	81	NM_032085.1
	Antisense(5'-3')	TTTTGTTTTGCTGGGGTTTC		
COL4A1	Sence(5'-3')	ATGCCAGGAAGAGCAGGAACT	131	NM_001135009.1
	Antisense(5'-3')	CGACTACCAGGAAAGCCAACTC		
COL4A2	Sence(5'-3')	CGAGAGGCGTCTCTGGATTC	200	XM_039095257.1
	Antisense(5'-3')	TGCGTAAGGTTCGCCTTTCT		
COL4A5	Sence(5'-3')	ACCTGGAGTTTCAGGCATTG	325	XM_006257313.2
	Antisense(5'-3')	AGGAATGCCATCTTGACCTG		
COL5A1	Sence(5'-3')	ACAGCGGTCCCTGACACACCT	199	NM_134452.2
	Antisense(5'-3')	GGGGCTGGGTCTGCTCCTCA		
LAMA3	Sence(5'-3')	ACCAGCTTTTTGTGGTGGAC	208	NM_001393748.1
	Antisense(5'-3')	ACGCAGATGTGATGCAGAAG		
LAMB1	Sence(5'-3')	CATTGGCTCCAGATGTGATG	200	XM_003750137.5
	Antisense(5'-3')	AGTACCCATGCTGGCAAAAC		
LAMC1	Sence(5'-3')	TCTTGGACCTTACAGCCCGT	237	NM_053966.3
	Antisense(5'-3')	GTGCACACCACTTCCTTTGTC		
HSPB2	Sence(5'-3')	TTCAAGAGGTGCTGCATACG	141	XM_017593852.2
	Antisense(5'-3')	TGTGGCAAGCAAACTCTGTC		
IL-1β	Sence(5'-3')	CCTTGTGCAAGTGTCTGAAG	124	NM_012675.3
	Antisense(5'-3')	TAAGGATTGCTTCCAAGCCC		
MMP13	Sence(5'-3')	TGGTCCCTGCCCCTTCCCTA	88	NM_012929.1
	Antisense(5'-3')	CCGCAAGAGTCACAGGATGGTAGTA		
ADAMTS5	Sence(5'-3')	GGGGTCAGTGTTCTCGCTCTTG	129	NM_198761.2
	Antisense(5'-3')	GCCGTTAGGTGGGCAGGGTAT		
TIMP3	Sence(5'-3')	AGCCGTTTTTGCGCTTTC	129	NM_012886.3
	Antisense(5'-3')	GCTAAATCATGTGCCATGTGT		
ACAN	Sence(5'-3')	GACCCAAACAGCAGAAACAGC	250	NM_022190
	Antisense(5'-3')	CTGTTATCGCCACTTTCCCG		
SOX9	Sence(5'-3')	CGTGTGGATGTCAAAGCAAC	191	NM_080403
	Antisense(5'-3')	TCTTGATGTGCGTTCTCTGG		
GAPDH	Sence(5'-3')	TGCACCACCAACTGCTTAGC	156	NM_017008.4
	Antisense(5'-3')	GGATGCAGGGATGATGTTCTG		
